# Serological testing for Lyme disease: current assays, diagnostic performance, and challenges

**DOI:** 10.1128/jcm.00322-26

**Published:** 2026-07-20

**Authors:** Lili Tao

**Affiliations:** 1 Department of Pathology, Microbiology and Immunology, Vanderbilt Health 482981, Nashville, Tennessee, USA; Cleveland Clinic, Cleveland, Ohio, USA

## Abstract

Lyme disease, caused by *Borrelia burgdorferi sensu lato*, remains the most common vector-borne infection in temperate regions of the Northern Hemisphere. Laboratory diagnosis relies primarily on serologic testing, as current direct detection methods generally lack sensitivity in routine clinical specimens. Current serological diagnosis of Lyme disease relies on a two-tiered testing algorithm. In the article by E. J. Horn, B. Menefee, A. M. Schotthoefer, G. Dempsey, et al. (J Clin Microbiol 64:e01187-25, 2026, https://doi.org/10.1128/jcm.01187-25), the authors further demonstrated that the modified two-tiered testing (MTTT) algorithm has increased sensitivity compared with the standard two-tiered testing (STTT) algorithm for detecting antibodies from early Lyme disease samples. Despite the improved sensitivity using MTTT, important limitations persist, including limited sensitivity in early infection and the inability to distinguish active from past infection. This commentary briefly summarizes the current landscape of Lyme disease serologic assays, outlines established and emerging diagnostic algorithms, and discusses key challenges that continue to impact diagnostic accuracy and clinical decision-making.

## COMMENTARY

Lyme disease is a multisystem infection caused by spirochetes within the *Borrelia burgdorferi sensu lato* complex, most commonly *B. burgdorferi sensu stricto* in North America, and *Borrelia afzelii* and *Borrelia garinii* in Europe and Asia. The disease is transmitted by Ixodes ticks.

If untreated, Lyme disease can progress through distinct clinical stages (1). In the early localized stage, erythema migrans (EM), an expanding erythematous skin lesion, sometimes with central clearing, occurs in approximately 70%–80% of cases in the United States. This lesion may be accompanied by nonspecific symptoms such as fever, fatigue, headache, and myalgia. Subsequent dissemination can involve the nervous system, heart, and joints. In endemic regions with potential tick exposure, the presence of a characteristic EM lesion is considered sufficient for clinical diagnosis (2).

Because direct detection methods, such as culture and molecular methods (e.g., PCR), lack sensitivity in blood and other easily obtained specimens, laboratory diagnosis relies heavily on serologic assays that detect host antibody responses. Serologic testing is particularly important in patients with nonspecific or disseminated manifestations; however, interpretation requires careful consideration of disease stage, immune kinetics, and pretest probability. Accordingly, the evolution of serologic testing algorithms reflects ongoing efforts to improve early detection while maintaining specificity.

## SEROLOGIC ASSAYS FOR LYME DISEASE

### Current recommended testing algorithms

#### The standard two-tiered testing

Despite the introduction of the first FDA-cleared serologic assay for Lyme disease in 1987, followed by the development of numerous additional assays, testing remained largely nonstandardized prior to 1995 (3). This lack of standardization resulted in substantial interlaboratory variability, as well as intra-laboratory inconsistency when the same specimens were tested repeatedly. A 1992 survey highlighted these issues, reporting that 55% of laboratories failed to detect antibodies in a case-defined serum sample, while 4% to 21% of laboratories failed to correctly identify strongly positive sera or immunoglobulin G-reactive samples (4).

In response to these challenges, the Centers for Disease Control and Prevention (CDC) recommended a standardized two-tiered testing (STTT) algorithm in 1995 following the Second National Conference on Serologic Diagnosis of Lyme Disease (5). This approach consists of an initial sensitive enzyme immunoassay (EIA) or immunofluorescence assay, followed, if positive or equivocal, by confirmatory western immunoblot testing.

The recommendation also standardized the interpretation criteria. An IgM immunoblot is considered positive if at least 2 of 3 bands (24, 39, and 41 kDa) are present, while an IgG immunoblot requires at least 5 of 10 specified bands (18, 21, 28, 30, 39, 41, 45, 58, 66, and 93 kDa). Importantly, IgM immunoblot results should only be interpreted in patients with ≤30 days of symptoms, as IgM antibodies have relatively poor specificity and may lead to misdiagnosis when used as a diagnostic criterion in patients with symptoms beyond the acute stage.

#### The modified two-tiered testing

Although STTT improved standardization, its reliance on immunoblotting introduces subjectivity and limits sensitivity, particularly in early infection. These shortcomings motivated the development of alternative strategies aimed at improving both testing efficiency and diagnostic performance.

In 2019, the U.S. Food and Drug Administration cleared the first modified two-tiered testing (MTTT) algorithm, and the CDC subsequently endorsed MTTT as an acceptable alternative (6). MTTT replaces the second-tier immunoblot with a second EIA, typically using distinct antigens from the first-tier assay.

Multiple studies have demonstrated that MTTT provides improved sensitivity, particularly in early Lyme disease, while maintaining comparable specificity to STTT (7–13). Gains in sensitivity are most pronounced in patients with early localized infection, including those with EM. In the current study by Horn et al., MTTT again demonstrated higher sensitivity than STTT for the serologic diagnosis of Lyme disease across different assays (14). Notably, MTTT identified a higher proportion of cases among patients presenting with erythema migrans, a hallmark of early infection.

In addition to increased sensitivity, MTTT offers improved standardization, reduced subjectivity, and compatibility with high-throughput automation, which may enhance cost-effectiveness and reproducibility (15).

## CHALLENGES IN SEROLOGICAL DIAGNOSIS OF LYME DISEASE

Despite its high sensitivity for detecting disseminated and late-stage Lyme disease and improved performance in early infection by MTTT, serologic testing remains fundamentally limited by its dependence on host antibody responses. During the early “window period,” which lasts for a few days or weeks following infection, antibodies may not yet be detectable, resulting in reduced sensitivity. IgG responses typically become reliably detectable several weeks after infection in untreated patients (7).

Although EM is considered diagnostic in endemic settings, laboratory confirmation remains important in several contexts. First, EM-like lesions can occur in other conditions, such as southern tick-associated rash illness. Second, EM presentations can be atypical; up to ~10% of cases may exhibit vesiculobullous, purpuric, or necrotic features (16). Third, a subset of patients presents without EM but with nonspecific systemic symptoms, in whom laboratory testing supports diagnosis.

The study by Horn et al. demonstrated that, even with MTTT, the sensitivity of serologic testing was only 32% among patients presenting with EM (14). Seropositivity increased with time from rash onset: antibodies were detected in 26%, 50%, and 100% of cases within 1 week, 2 weeks, and more than 2 weeks after EM onset, respectively. Together, these findings highlight the intrinsic delay in humoral immune responses and underscore the need for improved early diagnostic strategies.

## EMERGING SEROLOGIC APPROACHES

In response to the limitations of current algorithms, newer serologic approaches aim to improve early detection while simplifying testing workflows.

A novel single-tier serologic assay, termed the “Hybrid Lyme ELISA,” incorporates a microplate coated with recombinant VlsE and a soluble, ready-to-use horseradish peroxidase-conjugated C6 peptide to detect antibodies against *B. burgdorferi*. This integrated “two-in-one” design demonstrated substantially improved sensitivity for early Lyme disease compared with STTT and MTTT (94% versus 64% and 76%, respectively), while maintaining comparable specificity (17). This approach highlights an innovative strategy to consolidate the traditional two-tiered algorithm into a single assay format while improving early-stage detection. However, it is important to note that the reported sensitivities of STTT and MTTT in this study were higher than typically observed, underscoring the need for independent validation in broader clinical cohorts.

In addition to improving sensitivity, newer-generation assays aim to simplify the testing process. A synthetic peptide-based multiplexed vertical flow assay has been developed as a point-of-care test for Lyme disease. This assay employs a multiantigen panel composed of synthetic peptide immunogenic targets to detect both IgM and IgG antibodies, coupled with a machine learning-based diagnostic algorithm that interprets results from smartphone-captured images. Using samples from the CDC Lyme disease repository, the assay demonstrated 100% agreement with STTT and 96.6% agreement with MTTT. In a subset of immunoblot-positive early-stage Lyme disease samples, the assay achieved a sensitivity of 95.5% and a specificity of 100% (18). Although this study did not demonstrate a clear improvement in sensitivity compared with existing algorithms, the simplified workflow and point-of-care format highlight its potential to enhance accessibility and support timely clinical decision-making.

## PROGRESS IN OTHER DIAGNOSTIC METHODS

Given the inherent limitations of serology, complementary diagnostic approaches are being actively explored. Serologic testing alone cannot address all clinical scenarios, particularly in immunocompromised patients who may have impaired antibody responses, or in cases where distinguishing active from past infection is clinically important.

Molecular diagnostics offer the advantage of direct pathogen detection. While conventional PCR has limited sensitivity in blood, newer approaches such as droplet digital PCR (ddPCR) have improved analytical sensitivity, detecting as few as approximately three genome copies. Performance of ddPCR appears to be influenced by specimen processing, with improved sensitivity observed in low-speed centrifuged blood samples (e.g., 260 × *g* for 10 min). In a study with a limited number of clinical specimens, ddPCR demonstrated a sensitivity of 58.5% for early-stage Lyme disease, compared with 24.4% using STTT (19). This approach has also been extended to multiplex platforms for simultaneous detection of multiple tick-borne pathogens, with comparable analytical sensitivity for *B. burgdorferi* (20).

Cell-free DNA (cfDNA) sequencing represents another emerging strategy. In patients with laboratory-confirmed EM, cfDNA testing demonstrated a sensitivity of 64%, compared with 50% for MTTT. Notably, combining cfDNA with MTTT increased sensitivity to 86% (21). Although cost and availability currently limit clinical implementation, this approach may be particularly useful in diagnostically challenging cases.

Beyond direct detection methods, advances in immunologic profiling have enabled the identification of biomarkers that may help distinguish active from past infection. Proteome-wide microarray analyses have identified novel early antigen targets, such as BafPKo_A0001, BafPKo_D0016, and BafPKo_A0029, in *Borrelia afzelii*. Antibody responses to these antigens demonstrated improved sensitivity compared with C6 peptide-based assays and STTT, particularly when used in combination (22). Additionally, VlsE-specific antibody isotype profiling reveals stage-dependent immune signatures, which can be leveraged using machine learning models to predict disease stage (23). These approaches highlight a shift toward more nuanced, biology-driven diagnostics.

## CONCLUSIONS

Serologic testing remains the cornerstone of Lyme disease diagnosis, particularly in disseminated and late-stage infection. While modified two-tier algorithms have improved sensitivity and standardization, important limitations persist, especially during early infection. Emerging assays, including enhanced serologic platforms, molecular diagnostics, and host-response biomarkers, offer promising avenues to improve diagnostic accuracy. Future diagnostic strategies will likely integrate multiple modalities to overcome the limitations of individual assays. Until these approaches are fully validated and implemented, clinical judgment remains essential, and laboratory results must be interpreted within the appropriate clinical and epidemiologic context.
